# Lanthanide-Dependent Self-Assembly of Ion-Extractant Complexes at Liquid Interfaces: Inversion or Exclusion?

**DOI:** 10.1021/acs.jpcb.6c03936

**Published:** 2026-10-05

**Authors:** Pan Sun, Uvinduni I. Premadasa, David Walwark, Wei Bu, Benjamin Doughty

**Affiliations:** † Chemical Sciences Division, 6146 Oak Ridge National Laboratory, Oak Ridge, Tennessee 37831, United States; ‡ Ames National Laboratory, Iowa State University, Ames, Iowa 50011, United States; § NSF’s ChemMatCARS, Pritzker School of Molecular Engineering, University of Chicago, Chicago, Illinois 60637, United States

## Abstract

The separation of lanthanide elements by solvent extraction is traditionally governed by differences in the thermodynamic stability of extracted complexes; however, ion-specific interfacial complexation can play an important role in determining selectivity in kinetic separations. Nevertheless, the structural and chemical origins of such interfacial selectivity remain poorly understood. In this work, we combine sum frequency generation (SFG) vibrational spectroscopy with X-ray scattering to resolve interfacial structures of extractant–ion complexes at liquid surfaces. Measurements across pH and lanthanide concentration regimes reveal how changes in aqueous conditions alter the molecular organization of interfacial bis­(2-ethylhexyl) phosphoric acid (HDEHP)–lanthanide ion complexes. Across the lanthanide series, light lanthanide ions, e.g., those from La to Dy, tend to form relatively ordered monolayers with HDEHP as characterized by strong CH and weak OH vibrational signals with corresponding electron density profiles and surface ion concentrations. In contrast, heavy lanthanide ions, e.g., those from Er to Lu, tend to produce more disordered interfacial assemblies as reflected by weaker CH stretches, stronger OH signals that are accompanied by surprisingly low lanthanide ion surface densities. These systematic, structural, and chemical differences suggest that HDEHP–heavy lanthanide complexes are more readily organized into increasingly centrosymmetric structures that are poised for transport, explaining their apparent absence at liquid–liquid interfaces at equilibrium. These results establish interfacial organization as a key factor governing lanthanide extraction chemistry and point to kinetic handles to impart unconventional selectivity.

## Graphical abstract

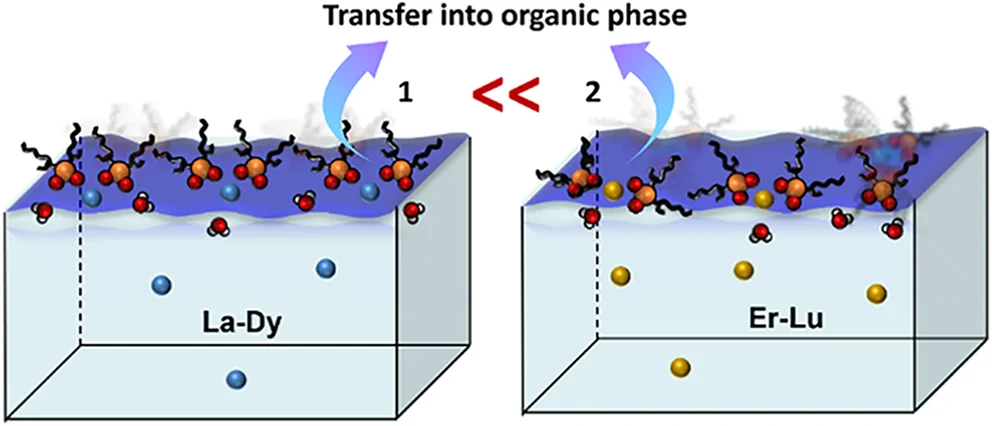

## Introduction

Solvent extraction is the workhorse process in the separation and purification of critical elements,^1–4^ including the technologically important family of rare-earth elements.^5–8^ In solvent extraction, target ions are complexed by amphiphilic ligands at liquid–liquid (L–L) interfaces and subsequently shuttled across the surface via thermodynamically downhill reactions. Among commonly used extractants, bis­(2-ethylhexyl) phosphoric acid (HDEHP) has received considerable attention because it binds strongly with trivalent metal ions and is broadly relevant to rare-earth extraction systems.^9–11^ While used at industrial scales, the structural and chemical origins of selectivity at interfaces, and potential kinetic handles to modulate selectivity, remain surprisingly poorly understood due to challenges in directly measuring interfacial phenomena.

Here, we note that in solvent extraction the interface plays a central role in defining mechanism as it is where metal ions first interact with extractant molecules before transferring into an organic phase.^12–16^ At this boundary, ion binding,^14,15^ ligand organization,^13,17^ solvation,^18^ and mass transport^16,19^ are closely connected. Directly probing these processes at L–L interfaces, however, is difficult because the interfaces are dynamic and multiple competing processes occur at the same time.^12,17^ Adding complexity, we note here that HDEHP is also slightly soluble in water.^16,20–22^ As a result, an interfacial layer can be formed via adsorption from either the organic phase or the aqueous phase, with the resultant interfacial structure depending strongly on solution conditions on both sides of the interface.

The air–liquid (A–L) interface offers a simpler system that makes it possible to focus on interfacial complexation and molecular organization without the added complexity of mass transport across two liquid phases;^14,15,20,21^ however, this simplification comes at the expense of insight into diluent effects and competitive solvation that ultimately mediate recognition and transport.^18,23^ Indeed, previous surface X-ray scattering studies have shown that HDEHP adsorbs to the air–water interface and that metal ions can accumulate beneath or within this interfacial layer.^20,21^ These studies confirmed the presence of an HDEHP monolayer-like structure, but they did not resolve how the ligands were organized or how ion binding changed the local interfacial environment. One reason is that the relatively short, branched alkyl chains of HDEHP provide limited structural contrast in X-ray measurements. Vibrational sum-frequency generation (SFG) spectroscopy is well suited to fill in these gaps given intrinsic sensitivity to molecular structure and charge at interfaces. In particular, CH stretches have been used to report on the ordering of the HDEHP tails, while OH stretches provide information about water and local electric fields from the charged phosphates that decorate the interface.^13,14,16^ SFG measurements have also been made at L–L interfaces, allowing for a comparison of idealized A–L surfaces with realistic competitive and time-evolving interactions that are present in active solvent extraction processes.^13,20,23,24^ As such, when A–L measurements are combined with measurements at L–L interfaces, new insight into interfacial complexation and binding can be inferred. Similarly, when combined with X-ray reflectivity (XR) and X-ray fluorescence near total reflection (XFNTR), SFG can help connect ligand ordering and water organization to ion adsorption at the interface.^14,25–28^

In this work, we examine how pH, lanthanide complexation, and chemical makeup affect the assembly and solvation of HDEHP at simplified A–L interfaces. We first use SFG to map how changes in pH and the presence of a representative lanthanide ion, Nd^3+^, influence ligand ordering and interfacial water structure. This insight is extended across a series of lanthanide ions with explicit comparison to new XR and XFNTR measurements probing interfacial structure and ion adsorption. The results show clear ion-dependent differences in the interfacial behavior of HDEHP. Light lanthanide ions are associated with surprisingly ordered interfacial complexes, whereas heavy lanthanide ions produce features consistent with increasingly disordered or more centrosymmetric structures, possibly inclusive of inverted-bilayer-like arrangements that were suggested in other reports.^14,15^ The current findings help clarify how lanthanide ions influence extractant organization at interfaces and provide insight into the interfacial processes that underlie lanthanide separations at large.

## Materials and Methods

### Reagents

LaCl_3_ (99.99%), NdCl_3_ (99.99%), GdCl_3_ (99.9%), DyCl_3_ (99.9%), ErCl_3_ (99%), LuCl_3_ (99.5%), and bis­(2-ethylhexyl) phosphoric acid, HDEHP (99%) were purchased from Sigma-Aldrich. Sodium hydroxide (NaOH) was purchased from Alfa-Aesar (98%) and hydrogen chloride (HCl) from Fisher Chemical (36.5 to 38.0%). All chemicals were used without further purification. Dihexadecyl phosphate, DHDP (99%) was purchased from Sigma-Aldrich and dissolved in the chloroform (99%, Sigma-Aldrich) to prepare 0.1 mM solution.

### Solution Preparations

HDEHP-aqueous solutions were prepared by mixing 2 g HDEHP with 100 mL pure water. The solutions were filtered to remove excess HDEHP, resulting in a saturated HDEHP-aqueous solution. The saturated HDEHP-aqueous solution was then diluted by a factor of 30 to prepare ∼10 μM HDEHP-aqueous phases in which lanthanide ions (concentration of 1.67 μM) were codissolved. Solution pHs were adjusted with HCl (0.01 M, 0.1 M) and NaOH (0.01 M, 0.1 M).

### Vibrational Sum Frequency Generation Spectroscopy (SFG)

Air–aqueous interfaces were supported in piranha-cleaned Teflon dishes; samples were equilibrated for 24 h before SFG measurements began. A custom-built system, as detailed elsewhere, was used to measure SFG spectra.^16,29,30^ In brief, broadband mid-infrared light centered at ∼3000 cm^–1^ and narrow-band near-infrared light centered near 803 nm, with a 1 nm (fwhm) were simultaneously focused onto the sample surface such that pulses overlapped both spatially and temporally. The beams impinged on the interface at an incidence angle of approximately 60° relative to the surface normal. The SFG response was collected in reflection, polarization resolved, dispersed, and detected using an Andor Newton CCD camera. Spectra were collected with an integration time of 3 min. In all cases, the measured SFG spectra were background-subtracted and normalized against the nonresonant signal from a gold film reference.

The measured SFG intensity is given by

1 $$I_{\mathrm{SFG}}\propto {\left|\chi_{\mathrm{NR}}^{\left(2\right)}e^{i\phi }+\sum_{q}\frac{A_{q}}{\omega_{\mathrm{IR}}-\omega_{q}+{i\Gamma }_{q}}\right|}^{2}$$

where χ_NR_^(2)^ denotes the nonresonant response, ω_IR_ is the frequency of the incident IR light, ω*_q_*, *A_q_* and Γ*_q_* are the resonant transition frequency, amplitude and line width of the *q*th-mode, respectively. The phase angle, φ, mixes in contributions from polarized bulk water via the χ^(3)^-effect that is included in χ_NR_^(2)^.^16,31,32^ Spectra were fit to eq 1 to extract parameters describing the assembly and structure of ligand-ion complex at the interface, as discussed in the text; all parameters are summarized in Tables S1–S3.

### X-ray Measurements

Synchrotron X-ray measurements were performed using a liquid surface scattering setup at beamline 15-ID of NSF’s ChemMatCARS, Advanced Photon Source, Argonne National Laboratory.^33^ The incident X-ray energy was 10 keV. A small Delrin trough was placed in a sealed helium-purged chamber to reduce background scattering from air and limit beam-induced damage. Prior to entering the chamber, the helium gas was humidified by passage through water. Data acquisition times were selected based on prior beam damage tests. To avoid repeated irradiation of the same location, the sample was translated horizontally between scans so that each measurement probed a fresh area of the surface. Analogous to SFG measurements, all samples were prepared ∼24 h before X-ray scattering measurements to ensure sample equilibration.

### X-ray Reflectivity

X-ray reflectivity (XR) data was collected as a function of the wave vector transfer normal to the interface, *Q*_*z*_ = |*k⃗*_s_ – *k⃗*_i_| = 2*k*_0_ sin α, where *k*_0_ = 2π/λ is the X-ray wavenumber, *k⃗*_i_ is the incident wave vector, and *k⃗*_s_ is the reflected wave vector.^34^ At each *Q*_*z*_, the specularly reflected intensity (*Q*_*xy*_ = 0) and the background intensity measured slightly off the specular condition (*Q*_*xy*_ ≠ 0, but small) were recorded simultaneously using a Pilatus 200 K area detector. Both signals were normalized to the incident beam intensity before analysis.

The XR data were fit to a model electron density profile, ρ­(*z*), along the surface-normal direction *z*, averaged over the interfacial *x*–*y* plane. The profile was described as a sum of error functions

2 $$\rho \left(z\right)=\frac{1}{2}\sum_{i=0}^{N}\mathrm{erf}\left(\frac{z-z_{i}}{\sqrt{2}\sigma }\right)\left(\rho_{i}-\rho_{i+1}\right)+\frac{\rho_{0}+\rho_{N+1}}{2}$$

where $\mathrm{erf}\left(z\right)=\frac{2}{\sqrt{\pi }}\int_{0}^{z}e^{-t^{2}}dt$ , *N* is the number of slabs, ρ_*i*_ is the electron density of the *i* ^th^ slab, ρ_0_ = ρ_water_ and ρ_*N*+1_ = ρ_air_ are the electron densities of bulk water and air, respectively, *z*_*i*_ is the position of the *i* ^th^ interface, and σ is the interfacial roughness. The thickness of the *i*th slab, *d*_*i*_, is defined as |*z*_*i*_ – *z*_*i*–1_|. Reflectivity curves were calculated from the electron density profile in eq 2 using the Parratt formalism after discretization.^34^ Additional fitting parameters included a *Q*_*z*_ offset to account for small instrumental misalignments and an overall scale factor. Fitting parameters are shown in Table S4.

### X-ray Fluorescence Near Total Reflection (XFNTR)

X-ray fluorescence near total reflection (XFNTR) measurements were performed by collecting fluorescence spectra over a series of *Q*_*z*_values near the critical wave vector transfer, *Q*_*c*_, for total reflection.^35^ A Vortex-60EX multicathode energy-dispersive X-ray detector positioned above and perpendicular to the interface was used to record the fluorescence spectra. Each spectrum was normalized to the incident beam intensity and corrected for detector dead time. After normalization, the lanthanide *L*_α1_ fluorescence peak was fitted with a Gaussian function, and the integrated peak area was used as the XFNTR signal. The XFNTR signal was modeled by integrating the X-ray intensity and the lanthanide ion concentration over the overlap volume defined by the X-ray beam path and the detector sensitivity region in the aqueous phase (Figure S2). The details have been described in previous work.^34,35^

## Results and Discussion

As mentioned above, the A–L interface provides a platform to study molecular adsorption and ion partitioning without the complexities of mass transport and oil-phase solvation. While omitting key insight into important diluent effects, the A–L interface does afford the ability to *infer* structural insight of would-be complexes, especially when compared to measurements made at L–L interfaces.^24^ Since HDEHP is slightly water-soluble, we prepared ∼10 μM HDEHP solutions (below its critical micelle concentration of 1.6 mM^38^) in various compositions (detailed in Materials and Methods).^20,21^ The concentration of 10 μM was selected to maintain a measurable interfacial population of HDEHP while avoiding bulk precipitation. In previously reported experiments, higher aqueous HDEHP concentrations led to the formation of insoluble HDEHP–lanthanide complexes after the addition of lanthanide ions.^18,22^ Such precipitation would complicate the interpretation of the SFG spectra by introducing bulk aggregates and reducing control over the interfacial species and ligand concentrations. This lower concentration was therefore chosen to isolate the interfacial complexation behavior under conditions where the solution remained macroscopically homogeneous. We acknowledge that these conditions do not reproduce the extractant concentration or phase composition of an operating solvent extraction system. Rather, the present experiments are intended as a simplified model for identifying ion-dependent differences in HDEHP–lanthanide complexation and interfacial organization. The results therefore provide molecular-level insight into possible interfacial structures and trends in lanthanide-dependent behavior, but they should not be interpreted as a direct representation of the complete equilibrium speciation or extraction performance under practical process conditions. Previously, the surface structures of these HDEHP-aqueous solutions have been probed with surface X-ray scattering techniques including XR and XFNTR^20^ where monolayer structures of HDEHP with metal ions were observed; however, the ordering, structure and specific interactions of metal ions with HDEHP were not elucidated due to limited contrast—this gap is first addressed by SFG measurements, the details of which can be found in the Materials and Methods section, probing the same interfaces.

Figure 1A,1B show SFG spectra from the surface of HDEHP decorated aqueous phases at equilibrium. Here we introduce two factors that affect the assembly and interactions of the ligands at the interface: (1) aqueous phase pH and (2) the addition of Nd^3+^ ions that partition to the surface. In all cases, the SFG spectra show three characteristic features: (1) methyl group symmetric stretching (SS) modes from CH_3_ groups at ∼2875 cm^–1^ with weak, unresolved signals from neighboring CH_2_–SS modes at 2850 cm^–1^ (2) CH_3_ Fermi resonances (FR) at ∼2950 cm^–1^ that are overlapped with the out-of-phase asymmetric CH_3_ vibrations (∼2965 cm^–1^) and (3) the broad OH stretching band extending from >3000 cm^–1^.^16^ In all cases, the interference between water and the CH signals results in asymmetric line shapes and apparent shifts in peak position that evolve depending on the relative phase.^31^ Quantitative analysis of the spectra requires fitting to isolate nascent molecular responses (details in the Materials and Methods section and tabulated in the Supporting Information).

**Figure 1:**
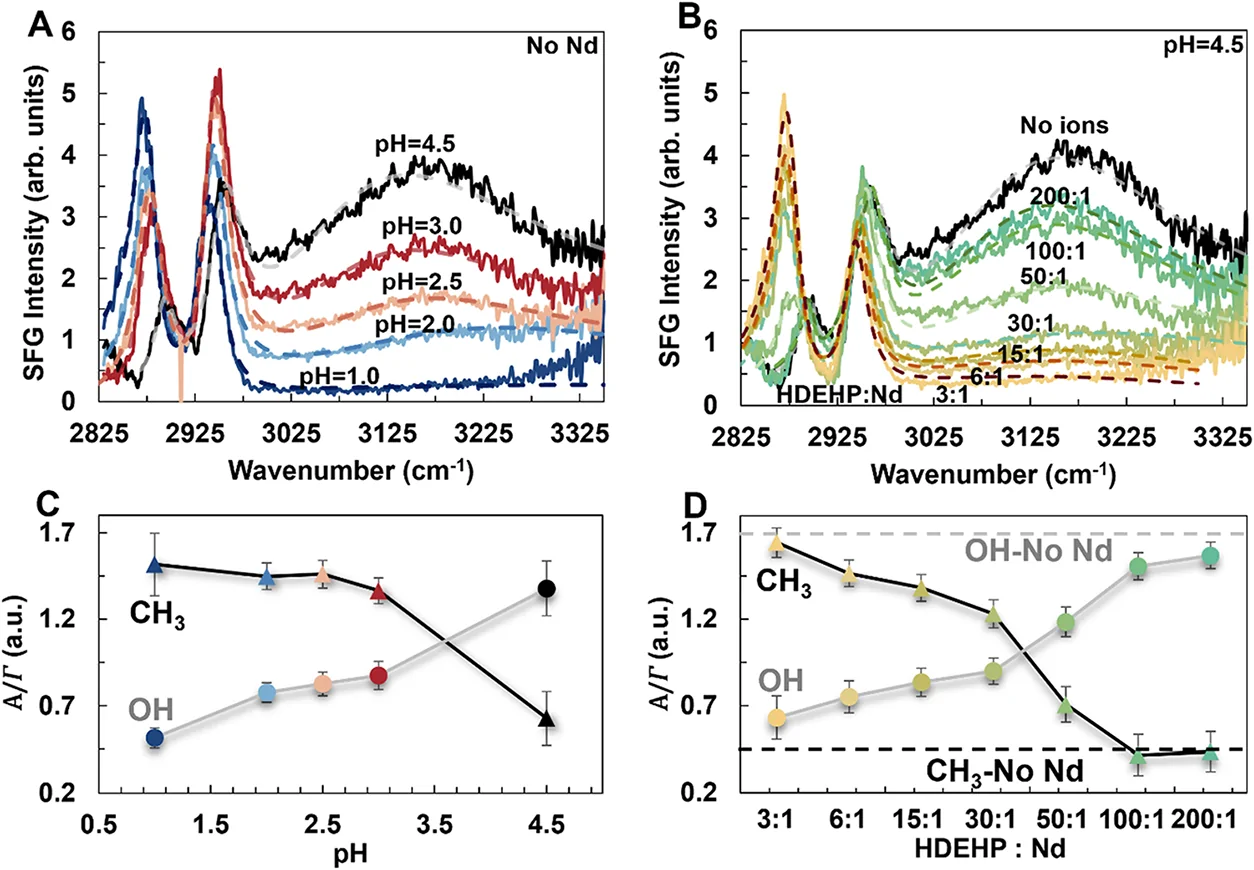
Effect of aqueous pH (A) and ratio of HDEHP to Nd (B) on the SFG spectra of HDEHP-aqueous solution. Variation of the CH_3_–SS and OH stretches intensity as a function of aqueous pH (C) and ratio of HDEHP to Nd (D). Dashed lines in (D) show signal levels in the absence of Nd for comparison.

As pH increases from 1.0 to 4.5, the CH_3_–SS band intensities plotted in Figure 1C decrease; suggesting a transition to a more disordered interface at elevated pHs. Simultaneously, the OH stretching signals increase as pH increases. These changes arise from the deprotonation of phosphate groups of HDEHP (p*K*_a_ ∼ 3.2).^39^ As such, at low pHs, hydrogen-bonding between ligands and water tends to yield more ordered surfaces (larger CH signals), whereas at higher pHs, the charged interface generates substantially more OH signals via static electric fields extending into the bulk.^31,40^ Surface tension measurements (Figure S3) indicate that decreasing the aqueous-phase pH lowers the surface tension. This trend is consistent with increased protonation of HDEHP and enhanced accumulation of HDEHP at the interface, in agreement with the corresponding increase in the CH signal observed by SFG. This physical picture is further confirmed by experiments where Nd^3+^ was introduced into the aqueous phase, as shown in Figure 1B. On addition of Nd^3+^, field aligned water signatures are suppressed due to charge-screening and ion interactions with the ligand headgroups (Figure 1D). Since one Nd^3+^ can bind with multiple ligands at the interface, the HDEHP reorganizes to yield a more ordered interface anchored by the hydrophilic Nd^3+^ ions in water.

Extending this physical picture to other lanthanide ions, we note that there is an expected variation based on the lanthanide contraction and associated solvation energies.^41,42^ Figure 2A,2B show the SFG spectra of ∼10 μM HDEHP aqueous solutions containing different lanthanide ions (as well as a control case with no additional ions) at pH 4.5. As before, SFG spectra were fit (Figure S1) and extracted amplitudes plotted in Figure 2C. Notably, the CH intensities change with Ln^3+^ identity with a trend toward increasingly disordered interfaces with increasing atomic number from La^3+^ to Dy^3+^, *i.e*., overall weaker CH_3_–SS signals and larger OH stretches. Notably, in the presence of heavier lanthanides, Er^3+^ and Lu^3+^, the spectral features more closely mimic systems containing no additional ions—an effect that, at L–L interfaces, was attributed to fewer interfacial complexes and the exclusion of heavy lanthanides at the interface.^24^ The changes in SFG response at the A–L interface can be explained by noting that

1. Heavy lanthanide ions might not strongly partition to the HDEHP monolayer at the A–L interface due to their strong solvation/coordination in the bulk phases.^20^
2. HDEHP-Ln^3+^ complexes at A–L interfaces can exist as centrosymmetric assemblies, in line with previous work suggesting the formation of a (partially) inverted bilayer.^14,15^

**Figure 2:**
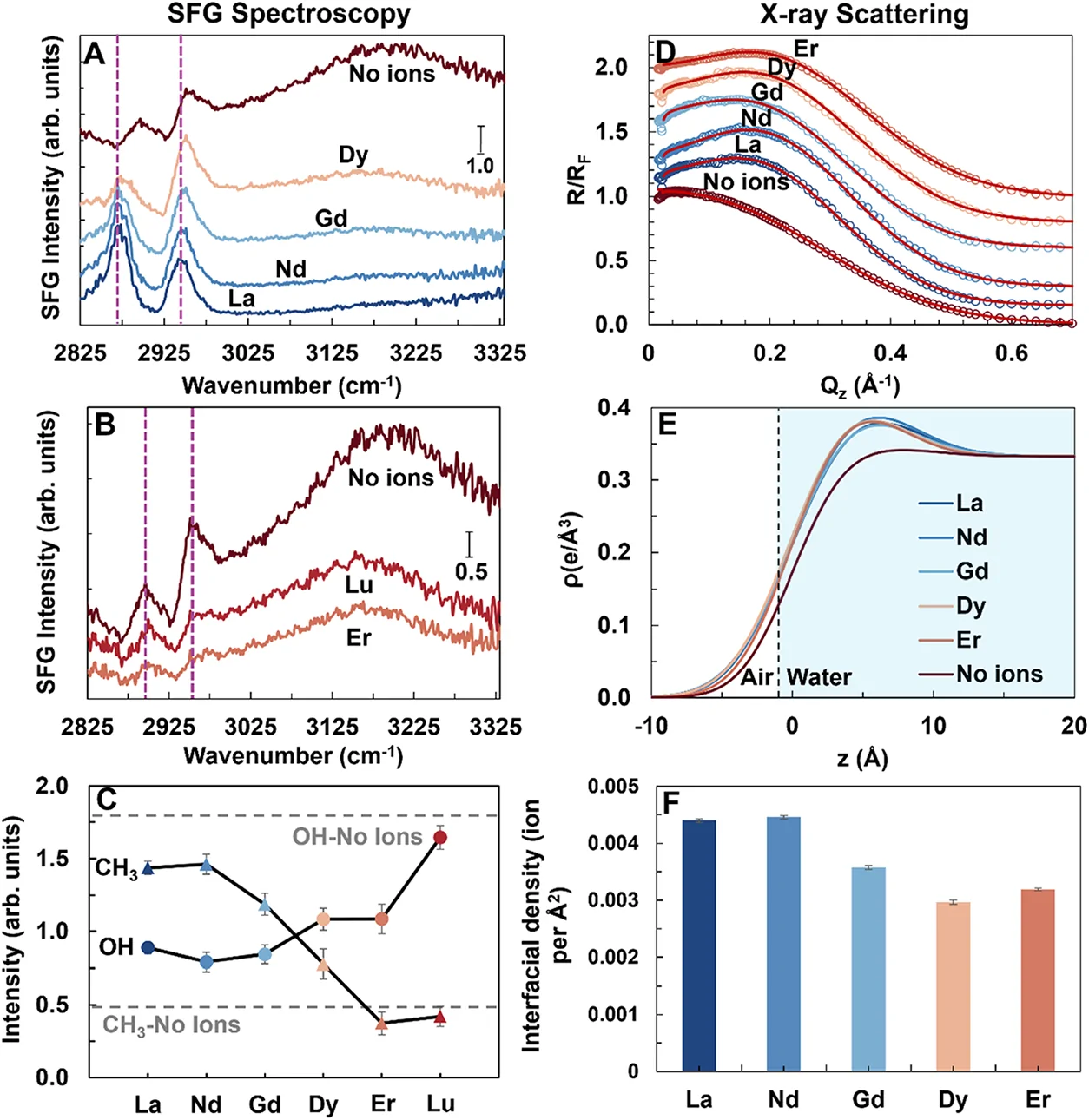
(A, B) Evolution of SFG spectra of HDEHP-aqueous solution containing various lanthanide ions (A: La to Dy, B: Er and Lu); (C) lanthanide dependent intensity of CH_2_ and OH stretches; (D, E) X-ray reflectivity measurements for HDEHP-aqueous solution containing various lanthanide ions and corresponding electron density profiles; (F) Intensity of different lanthanides at surface of HDEHP-aqueous solution measured by XFNTR. Spectra are vertically offset in A/B/D. Concentrations of lanthanide ions in the HDEHP-aqueous solution are 1.67 μM.

To differentiate between these possibilities, we characterized the interfacial structure and surface density of various lanthanide ions using synchrotron-based X-ray scattering, as shown in Figure 2D,E. X-ray reflectivity probes the electron density contrast across the A–L interface, providing an electron density profile that contains contributions from HDEHP, lanthanide ions, and water.^17^ As illustrated in Figure 2D, the presence of lanthanide ions alters the reflectivity, which is more clearly reflected in the corresponding electron density profiles shown in Figure 2E. Compared to the case of no lanthanide ions added, the addition of lanthanide increases the electron density near the water surface which is attributed to the headgroup of HDEHP coordinating with lanthanide ions. Notably, a strong element dependent difference was not observed in the electron density profiles indicating the presence of Ln^3+^ ions at the A–L interface in all cases.

To quantify the density of different lanthanide ions at the surface, we measured the X-ray Fluorescence Near Total Reflection (XFNTR) from the water surface, which directly reports on the surface density of lanthanide ions.^34,35^ As shown in Figure 2F, the surface density of lanthanide ions decreases by about 25% from La to Er. This observation differs from X-ray measurements of *insoluble monolayers* with long straight chained surfactants where enrichment of heavy ions was observed and linked to preferential extraction tendencies. This difference in observation can be explained by the distribution of HDEHP at the interface vs the bulk. Here, it is known that heavy Ln^3+^ ions can be held-back by aqueous-phase bulk aggregates formed with HDEHP.^16,20,21^ As such, the observed lower interfacial concentration of heavy Ln^3+^ ions is expected based on strong coordination/solvation with the slightly water-soluble extractants in the bulk aqueous phase. These observations are also in agreement with recent SFG measurements at L–L interfaces, where heavy lanthanides were found to be excluded from the interface following back extraction, owing to their stability in the respective bulk phases.^24^

In addition, the surface tensions of HDEHP-solutions containing different lanthanides (Figure S3) increase from 58 to 68 mN/m as the lanthanide varies from La to Lu, which can be attributed to two effects: (1) a decrease in the amount of HDEHP–lanthanide complexes adsorbed at the interface, and/or (2) changes in the intermolecular organization of the adsorbed complexes. As demonstrated in previous work, dissolved HDEHP can form aggregates with heavier lanthanides in the bulk aqueous phase.^20,21^ Such bulk aggregation could reduce the concentration of HDEHP–lanthanide complexes available for adsorption at the interface and thereby contribute to the higher surface tension observed for the heavier lanthanides. However, our measurements indicate that the interfacial population of HDEHP–heavy-lanthanide complexes is only approximately 25% lower than that of the corresponding light-lanthanide complexes. This relatively modest difference in interfacial population may not fully account for the increase in surface tension from approximately 52 to 68 mN/m. We therefore consider changes in the organization of the adsorbed HDEHP–lanthanide complexes to be an additional contributor to the surface-tension trend.

To test the idea whether heavier lanthanides form increasingly centrosymmetric structures with HDEHP at A–L interfaces, we turn to a linear chain organophosphorus analog: dihexadecyl phosphate (DHDP), where it is known that *inverted* bilayers spontaneously form at A–L surfaces in the presence of heavy lanthanide ions (Dy to Lu).^14,15^ To rationalize our HDEHP results, we measured SFG spectra of DHDP monolayers on the top of water containing Lu^3+^ as shown in Figure 3A. DHDP monolayers on a pure water phase yields similar spectral features as HDEHP decorated interfaces with analogous spectral assignments. Notably, the tails of DHDP are very ordered due to the linear structure and close packing (area per molecule is ∼40 Å^2^/molecule). An inverted bilayer of DHDP-Lu^3+^ complexes was generated using 0.1 mM Lu^3+^ following previous reports.^14,15^ From the resulting SFG spectra, it is apparent that CH signals decrease significantly while corresponding OH signals disappear almost entirely, consistent with the formation of a symmetric inverted bilayer. By decreasing the Lu^3+^ concentration to 1.67 μM, we can obtain a mixture of idealized DHDP monolayers and DHDP-Lu^3+^ inverted bilayer responses. Here, we note that the OH and CH signals describe complementary aspects of the interfacial structure and therefore must be considered together. The formation of a centrosymmetric assembly would reduce the CH signal because the vibrational responses from oppositely oriented alkyl chains partially or completely cancel. In contrast, a decrease in the OH signal primarily arises from changes in charge density, local H-bonding and solvation; as such, a decrease in the OH signal, by itself, does not provide evidence of a centrosymmetric structure. For example, adsorption of trivalent metal ions to a negatively charged DHDP monolayer can neutralize the interfacial charge and reduce the OH response from charge polarized water molecules.^14,25^ At the same time, metal–surfactant interactions can promote more ordered packing of the alkyl chains, producing an enhanced CH signal, as shown in the case of Nd in Figure 3A. Thus, a reduced OH intensity may be accompanied by an increase in alkyl-chain order.

**Figure 3:**
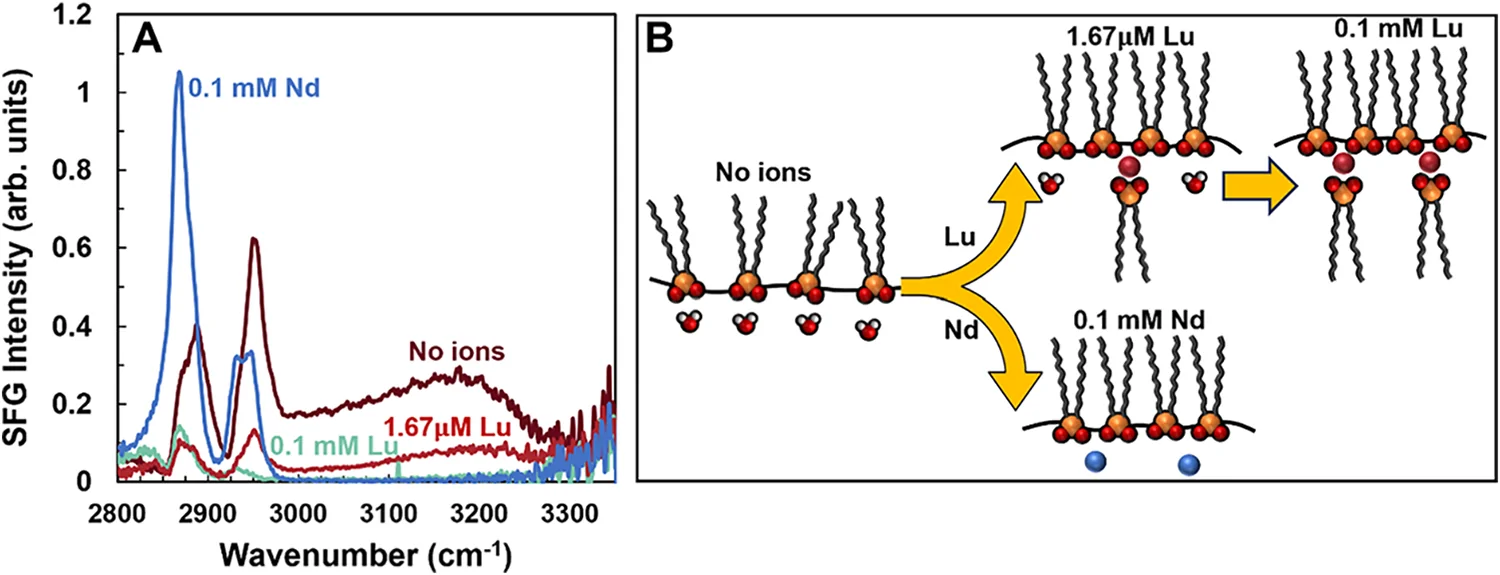
(A) Evolution of SFG spectra of DHDP monolayer on the surface of aqueous solution containing different concentrations of LuCl_3_ and NdCl_3_. (B) Illustration of structure evolutions for DHDP-Lu and DHDP-Nd complexes at interfaces.

In the HDEHP system, the lighter lanthanides exhibit larger CH signals together with reduced OH signals. We interpret this combination as increased ordering of the HDEHP alkyl chains accompanied by dehydration of the headgroup region, rather than formation of a centrosymmetric assembly. In contrast, the heavier lanthanides exhibit *weaker* CH signals, indicating either a more disordered arrangement or greater orientational cancellation of the alkyl chains. Their OH response reflects the corresponding changes in interfacial water structure. It was previously surmised that these symmetric complexes should be poised for extraction given their increasingly hydrophobic corona, which was why no such signals arising from heavy Ln^3+^ ions were observed at L–L interfaces after reaching equilibrium.^24^

The complementary results from SFG, X-ray scattering and surface tensions help clarify how the structure of HDEHP–ion complexes at the A–L surface depends on the identity of the lanthanide ion. As shown in Figure 4, light lanthanide ions, from La to Dy, form relatively well-organized monolayers with HDEHP at the water surface. This is evidenced by strong CH_3_ signals and weak OH signals observed by SFG together with the electron density profiles of the HDEHP–ion complexes and the surface densities of lanthanide ions obtained from X-ray scattering as well as lower surface tension. In contrast, heavy lanthanide ions, from Er to Lu, tend to form more disordered interfacial structures with HDEHP. This is evidenced by the weaker CH_3_ signal and stronger OH signal in SFG, as well as by the electron density profiles and surface ion densities derived from X-ray scattering with correspondingly higher surface tensions.

**Figure 4:**
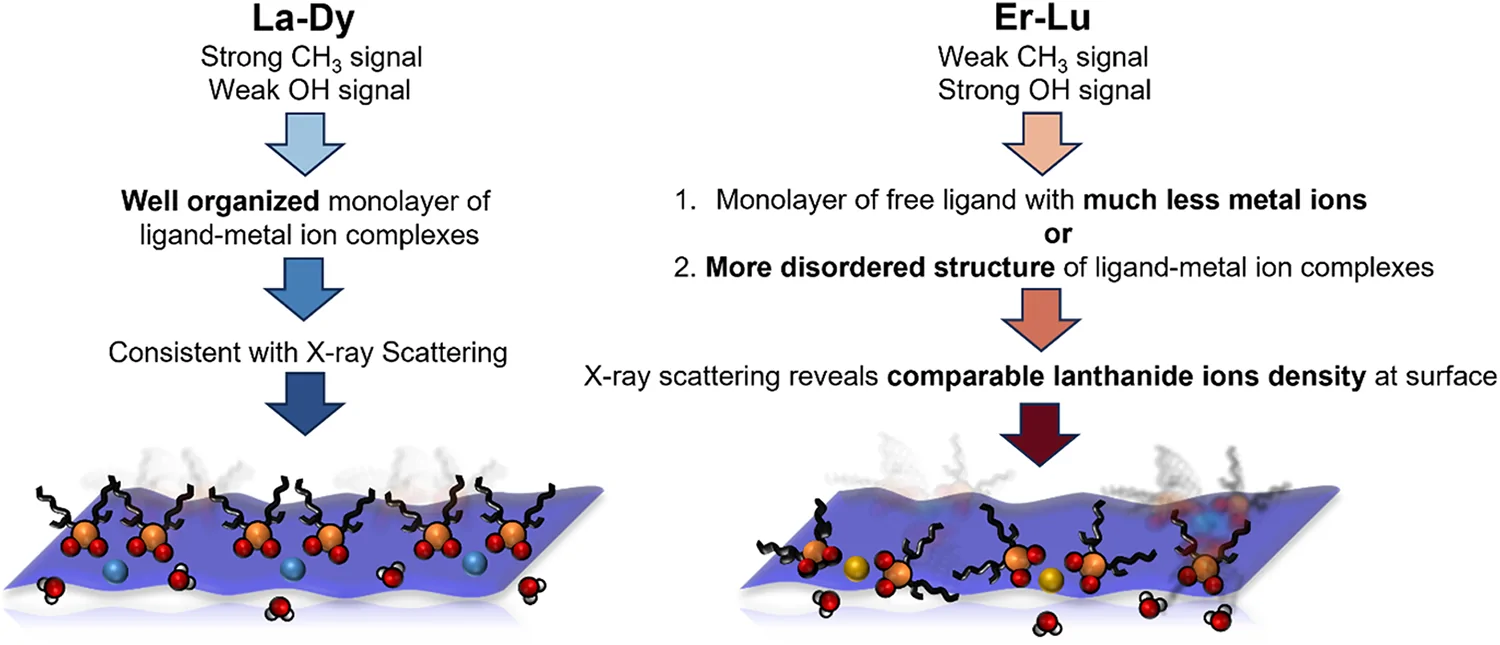
Illustration of interfacial structures of HDEHP-lanthanide complexes (Left: La–Dy; Right: Er–Lu).

Other mechanisms may also contribute to the substantial changes in interfacial organization observed across the lanthanide series in the present work. For example, a highly curved or locally deformed interface, in which surfactant molecules adopt opposing orientations across regions of strong curvature, could potentially generate similar spectroscopic signatures.^43^ Similar concepts have been proposed for extractant-containing liquid–liquid interfaces, where local curvature and interfacial deformation may accompany metal-ion complexation and transport. In particular, previous work by Schlossman and co-workers proposed a budding-type mechanism for the transport of trivalent metal ions across liquid–liquid interfaces,^17^ in which branched extractants such as HDEHP can promote curved local interfacial structures that facilitate transfer into the organic phase. Such a mechanism provides a useful conceptual comparison for understanding how HDEHP–metal complexes may adopt heterogeneous interfacial arrangements.

However, the present measurements were performed at an air–liquid interface, where there is no extraction-driven transfer into a second liquid phase. Recently, Clark et al. applied capillary wave theory to explain the interfacial structure at surfactant-laden oil–water interfaces.^44^ Accordingly, higher surface tensions, as measured in this work, suppress interfacial height fluctuations and high-curvature structures. Therefore, at the air–liquid interface, curvature-driven interpretations describing the data can be largely neglected, suggesting that the reduced SFG response is more plausibly attributed to changes in interfacial molecular organization, including increased disorder and partial inversion to form increasingly centrosymmetric arrangements of the HDEHP–metal complexes.

However, the current data does not provide conclusive evidence that HDEHP–heavy-lanthanide complexes adopt the centrosymmetric inverted-bilayer structure previously reported for the DHDP system.^14,15^ Therefore, we hesitate to assign a single discrete structural motif based on the present experimental evidence; highlighting instead that these observations are also consistent with a distribution of disordered or partially organized ligand–metal assemblies. Such structurally disordered HDEHP–ion complexes may be primed for transfer into the organic phase, consistent with previous observations at buried liquid–liquid interfaces.^24^

## Conclusion

Using surface-sensitive sum frequency generation spectroscopy and liquid-surface X-ray scattering, we demonstrate that both lanthanide identity and aqueous pH strongly influence the A–L interfacial structure of HDEHP–ion complexes. Light lanthanide ions form relatively ordered monolayers with HDEHP at the water surface, whereas heavy lanthanide ions give rise to more disordered interfacial assemblies. The preferential adsorption of light lanthanide ions over heavy lanthanide ions at the interface is consistent with previous X-ray measurements on the same system, in which the ligands are distributed between the interfacial region and the bulk aqueous phase, yet differ entirely from conclusions made by probing insoluble monolayer interfaces. These results demonstrate that without additional context into the functional L–L interface, connections between apparent structure and populations measured at model A–L interfaces may not map to solvent extraction at all. Here, these structural differences, as determined by SFG, provide a molecular-level explanation for extraction selectivity,^45^ particularly the preferential kinetic extraction of heavy lanthanides,^46^ where less ordered interfacial complexes more readily reorganize into transferable species, consistent with measurements at L–L interfaces and solvent extraction results. These results broadly highlight the importance of interfacial structure in solvent extraction and provide useful insight into the future design of more selective lanthanide separation systems.

## Supplementary Material



## Notes

Details of the SFG spectral fitting, X-ray reflectivity, and XFNTR analyses, as well as the aqueous pH- and lanthanide-dependent surface tension measurements of HDEHP at the aqueous interface (PDF)
