# Supplementary material for: Lanthanide-Dependent Self-Assembly of Ion-Extractant Complexes at Liquid Interfaces: Inversion or Exclusion?
Source: J Phys Chem B. 2026 Oct 5;130(41):11002–10. doi: 10.1021/acs.jpcb.6c03936 (PMC13679043; doi:10.1021/acs.jpcb.6c03936)
Supplement: Supplementary file 1 [file jp6c03936_si_001.pdf]

Lanthanide-Dependent Self-Assembly of Ion-Extractant Complexes at Liquid Interfaces: Inversion or Exclusion?

Pan Sun[1,2*], Uvinduni I. Premadasa[1], David Walwark Jr[3], Wei Bu[3], Benjamin Doughty[1*]

1. Chemical Sciences Division, Oak Ridge National Laboratory, Oak Ridge, Tennessee 37831, United States

2. Ames National Laboratory, Iowa State University, Ames, Iowa 50011, United States

3. NSF's ChemMatCARS, Pritzker School of Molecular Engineering, University of Chicago, Chicago, Illinois 60637, United States

*Corresponding authors: pans@ameslab.gov, doughtybl@ornl.gov

1. Fitting of SFG spectra

For several spectra, including pure water at pH 4.5 and aqueous solutions containing Er and Lu, the OH stretching band exhibits a strong intensity that overlaps with and interferes with the neighboring CH_3 stretching region. This spectral overlap makes fitting the full spectral range challenging. To reduce the risk of overfitting, the OH stretching frequency was fixed at 3100 cm^{-1} during the fitting procedure. This constraint results in an *apparent* blue shift of the fitted CH_3 stretching frequency for spectra with strong OH contributions, as reported in Tables S1–S3.

When the OH stretching frequency is allowed to vary freely, the fitted CH_3 stretching frequency is found to be consistent and in the expected ~2875 cm^{-1} range. However, this free fit approach cannot simultaneously fit spectra that have weak and strong OH signals using the same model, making direct comparison of OH-related parameters across samples difficult. Therefore, fixing the OH stretching frequency provides a more consistent fitting strategy that allows comparison across the full set of spectra while minimizing overfitting.

Table S1. Fitting parameters for SFG spectra of HDEHP-solution at different aqueous pH

pH	ω_1, cm^{-1}	A_1	Γ_1	ω_2, cm^{-1}	A_2	Γ_2	ω_3, cm^{-1}	A_3	Γ_3	Phase	NR
1.0	2876 ± 0.3	22.1 ± 1.3	14.6 ± 1.5	2941 ± 0.6	19.6 ± 1.1	14.7 ± 0.8	3100[a]	89.9 ± 9.6	174.3 ± 6.7	10.5 ± 2.8	0.4 ± 0.1
2.0	2876 ± 0.4	24.2 ± 1.1	16.7 ± 0.5	2941 ± 0.4	20.3 ± 0.6	14.5 ± 0.4	3100	224.3 ± 12.6	288.3 ± 13.1	13.6 ± 3.5	0.3 ± 0.1
2.5	2878 ± 0.4	24.4 ± 1.1	16.7 ± 0.6	2942 ± 0.4	23.2 ± 0.6	14.8 ± 0.4	3100	142.5 ± 9.7	172.3 ± 7.9	15.5 ± 2.8	0.6 ± 0.2
3.0	2879 ± 0.6	24.3 ± 1.0	17.8 ± 0.6	2943 ± 0.5	22.3 ± 0.6	15.0 ± 0.3	3100	124.6 ± 10.1	142.5 ± 6.4	18.8 ± 4.6	0.8 ± 0.1
4.5	2893 ± 1.2	8.1 ± 1.6	12.9 ± 1.9	2953 ± 1.2	19.7 ± 1.5	20.6 ± 1.2	3100	213.3 ± 22	154.8 ± 8.1	19.6 ± 5.3	0.9 ± 0.1

[a] The wavenumber of OH stretch (ω_3) was fixed at 3100 cm^{-1} to reduce the overfitting of spectra.

HDEHP:Nd	ω_1, cm^{-1}	A_1	Γ_1	ω_2, cm^{-1}	A_2	Γ_2	ω_3, cm^{-1}	A_3	Γ_3	Phase	NR
No ions	2897 ± 1.0	4.2 ± 0.7	8.9 ± 1.6	2959 ± 0.6	20.1 ± 1.8	22.8 ± 1.5	3100[a]	275.8 ± 10.4	163.6 ± 4.8	28 ± 6.5	0.7 ± 0.1
3:1	2874 ± 0.3	26.1 ± 1.2	15.8 ± 0.5	2943 ± 0.4	14.6 ± 0.9	13.9 ± 0.7	3100	184.2 ± 26.8	292.6 ± 40.1	9.6 ± 2.1	0.2 ± 0.1
6:1	2873 ± 0.4	23.3 ± 1.1	15.9 ± 0.5	2946 ± 0.4	14.5 ± 0.8	13.8 ± 1.0	3100	196.3 ± 17.4	261.2 ± 22.5	12.3 ± 3.2	0.2 ± 0.1
15:1	2874 ± 0.3	22.3 ± 1.0	16.1 ± 0.5	2947 ± 0.5	15.4 ± 0.8	14.3 ± 0.6	3100	196.6 ± 13.9	234.6 ± 16.1	15.2 ± 2.5	0.3 ± 0.1
30:1	2873 ± 0.4	21.4 ± 1.1	17.3 ± 0.7	2949 ± 0.4	16.2 ± 0.8	14.8 ± 0.6	3100	194.3 ± 12.3	216.2 ± 11.9	17.35 ± 3.7	0.3 ± 0.1
50:1	2881 ± 0.8	11.6 ± 1.3	16.5 ± 1.6	2954 ± 0.6	18.7 ± 1.2	17.2 ± 0.9	3100	221.2 ± 12.2	186.6 ± 8.6	20.3 ± 5.8	0.4 ± 0.1
100:1	2895 ± 1.1	3.6 ± 0.8	8.8 ± 1.8	2959 ± 0.7	19.8 ± 1.3	19.8 ± 1.2	3100	174.3 ± 5.6	163.6 ± 4.8	23.5 ± 9.8	0.5 ± 0.1

200:1	2886 ± 1.0	3.5 ± 0.6	8.1 ± 1.5	2960 ± 0.6	19.4 ± 1.7	21.8 ± 1.4	3100	265.6 ± 10.1	169.2 ± 5.3	25.7 ± 12.1	0.5 ± 0.1

Table S2. Fitting parameters for SFG spectra of HDEHP-solution containing different ratio of HDEHP to Nd^{3+}

[a] The wavenumber of OH stretch (ω_3) was fixed at 3100 cm^{-1} to reduce the overfitting of spectra.

Table S3. Fitting parameters for SFG spectra of HDEHP-solution containing different lanthanides

	ω_1, cm^{-1}	A_1	Γ_1	ω_2, cm^{-1}	A_2	Γ_2	ω_3, cm^{-1}	A_3	Γ_3	Phase	NR
No ions	2895 ± 1.3	5.1 ± 1.1	10.8 ± 2.1	2957 ± 1.5	15.8 ± 1.4	23.1 ± 1.6	3100[a]	255.8 ± 18.6	144.5 ± 5.7	28 ± 6.5	0.8 ± 0.1
La	2875 ± 0.3	23.3 ± 0.6	16.3 ± 0.3	2945 ± . 5	18.1 ± 0.6	16.2 ± 0.5	3100	211.2 ± 6.3	237.6 ± 7.9	6.7 ± 2.5	0.2 ± 0.1
Nd	2873 ± 0.4	23.9 ± 0.9	16.4 ± 0.5	2946 ± 0.4	15.9 ± 0.7	14.3 ± 0.6	3100	160.6 ± 9.8	202.9 ± 11.8	5.6 ± 1.5	0.3 ± 0.1
Gd	2873 ± 0.5	20.4 ± 1.1	17.2 ± 0.7	2948 ± 0.5	15.4 ± 10.8	14.5 ± 0.6	3100	155.1 ± 9.3	183.8 ± 9.2	6.8 ± 2.7	0.4 ± 0.1
Dy	2877 ± 0.8	13.5 ± 1.3	17.3 ± 1.5	2953 ± 0.6	18.9 ± 1.1	17.1 ± 0.8	3100	173.6 ± 9.6	160.2 ± 6.8	10.1 ± 3.5	0.4 ± 0.1
Er	2895 ± 2.3	12.2 ± 2.1	32.7 ± 4.2	2970 ± 2.6	12.8 ± 3.6	42.1 ± 6.7	3100	170.1 ± 13.9	156.6 ± 7.3	18.5 ± 4.5	0.5 ± 0.2
Lu	2902 ± 1.2	5.7 ± 0.7	13.7 ± 1.6	2969 ± 1.5	112.7 ± 2.6	34.5 ± 4.2	3100	282.6 ± 11.7	171.7 ± 5.2	25 ± 10.3	0.4 ± 0.1

[a] The wavenumber of OH stretch (ω_3) was fixed at 3100 cm^{-1} to reduce the overfitting of spectra.

Table S4. Fitting parameters from X-ray reflectivity measurements[a]

	d (Å)	ρ (e^-/Å^3)	σ (Å)
No ions	9.9 ± 1.6	0.346 ± 0.102	2.98 ± 0.21
La	7.8 ± 1.3	0.427 ± 0.105	3.58 ± 0.19
Nd	7.9 ± 1.3	0.432 ± 0.082	3.42 ± 0.16
Gd	7.3 ± 1.2	0.429 ± 0.123	3.54 ± 0.13
Dy	6.6 ± 1.5	0.453 ± 0.120	3.46 ± 0.22
Er	7.1 ± 1.4	0.425 ± 0.061	3.14 ± 0.18

[a] Parameters: layer thickness d; layer electron density ρ; interfacial roughness σ.

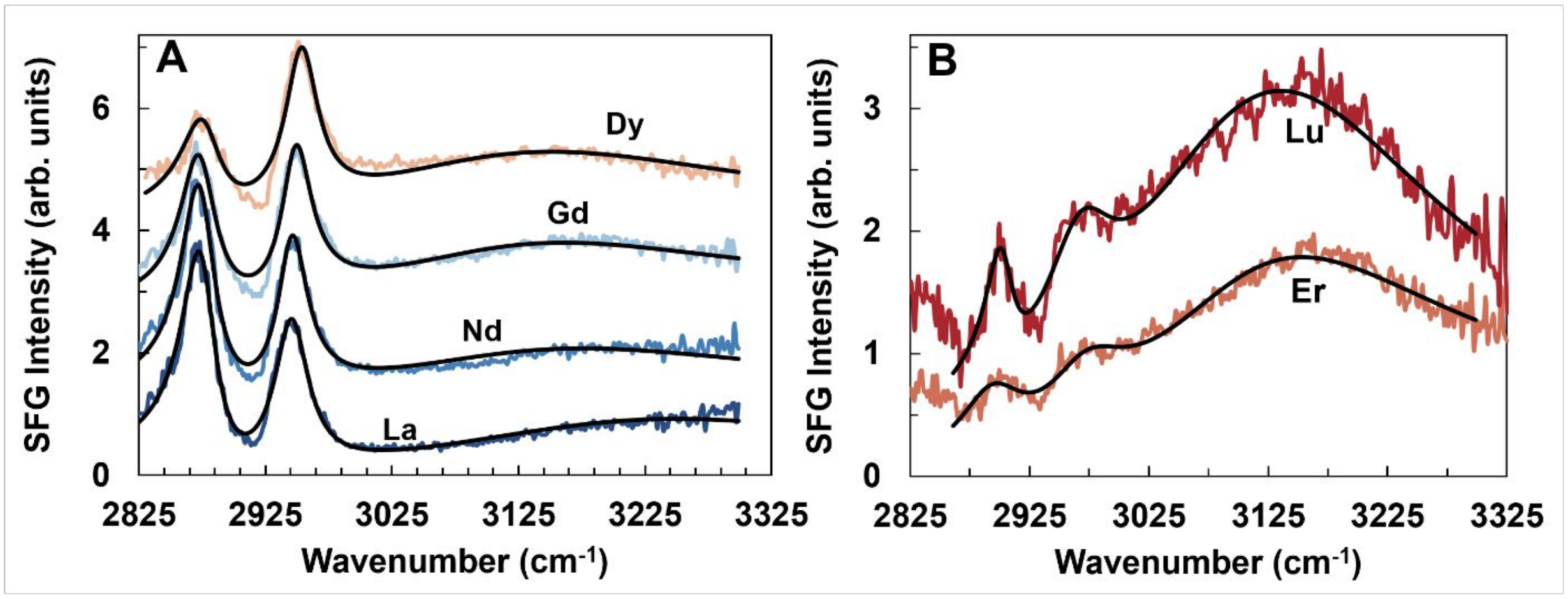

Figure S1. Fitting of SFG spectra from surface of HDEHP-solution containing different lanthanide ions (A: La, Nd, Gd, Dy; B: Er, Lu)

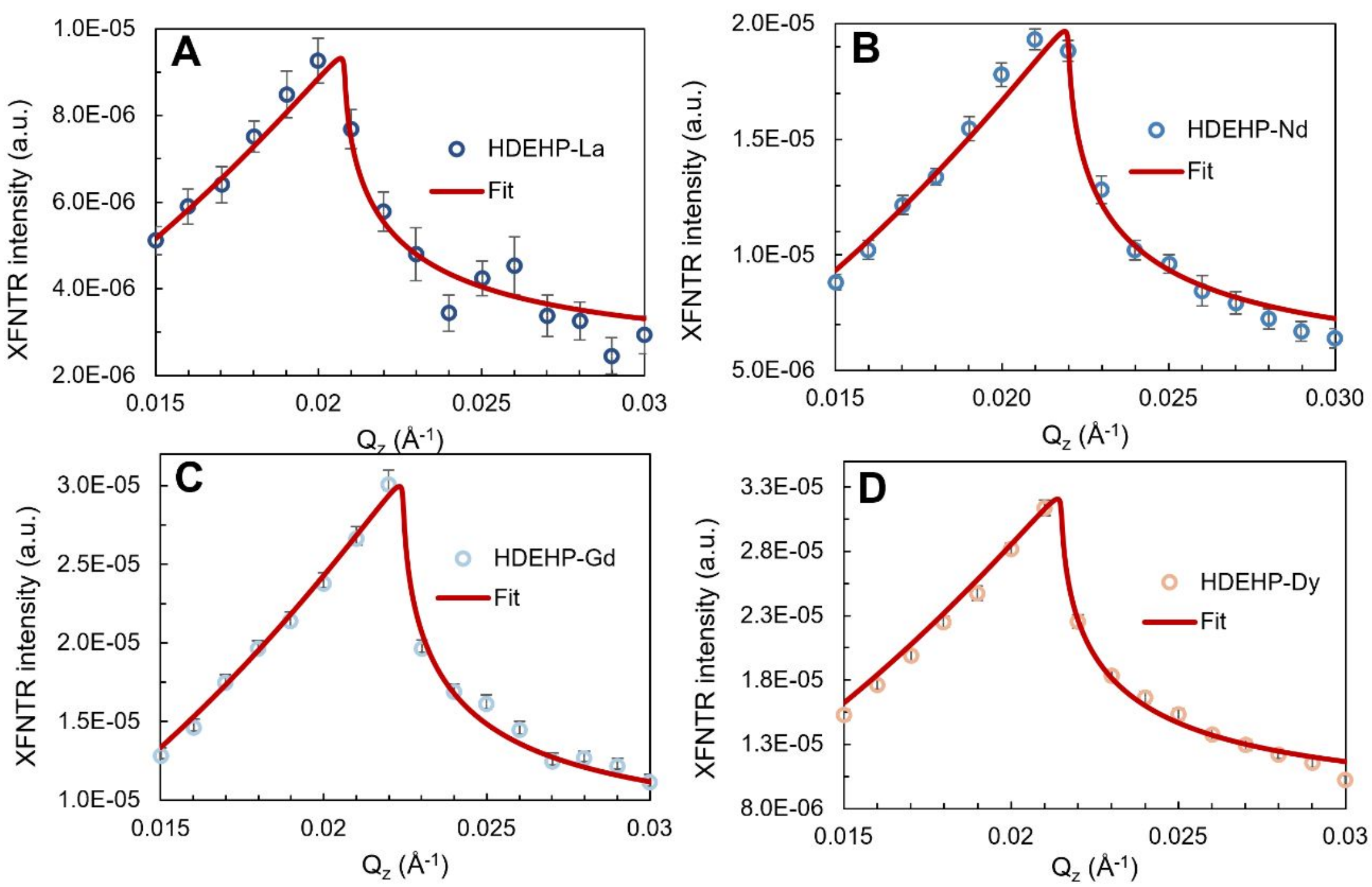

Figure S2. XFNTR of surface of HDEHP-solution containing different lanthanide ions. (A-D: La, Nd, Gd, Dy)

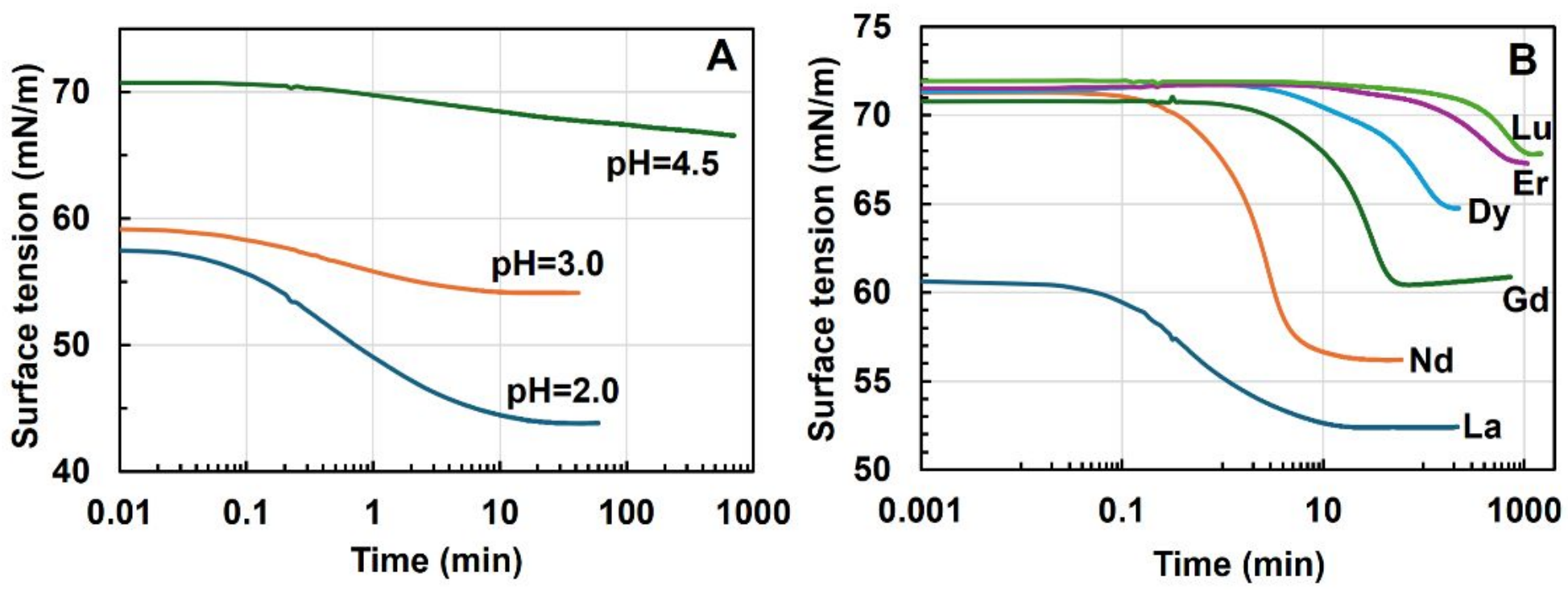

Figure S3. Surface tension of HDEHP-aqueous solution at different pH (without lanthanides) (A) and containing different lanthanide ions (aqueous pH of 4.5) (B).
